# Methods and Materials to Be Used in Filling Teeth

**Published:** 1873-05

**Authors:** J. A. Stipp


### METHODS AND MATERIALS TO BE USED IN FILLING TEETH.

READ BEFORE THE MISSISSIPPI VALLEY DENTAL ASSOCIATION, BY J. A. STIPP, D. D. S.

The subject under consideration is one that has not been passed without attention from the profession, but has called forth not a little study and thought. It has been with many a subject of life-long experiment and hard labor.

The methods, materials, and devices employed at the present day, for the purpose of filling teeth are quite numerous; so much so, that an attempt to describe the various substances, and define their respective qualities, would be a task far beyond anything contemplated in this paper.

But I will endeavor to confine myself to the subject, and consider in part that only which is the *best* and most suitable for the purpose of filling teeth.

In the selection of materials for filling teeth, there are some very important considerations that should constantly be kept in view. The first and principal of which is the protection of the tooth from further decay. This is to be done by protecting the affected part from the influence of those agencies upon which the disease depends.

Meterials should be selected that will not under any circumstances operate either as a local or constitutional injury, and to meet this consideration its properties should be well understood. Any substance, whether simple or compound, that has not the power of retaining its identity and integrity while subjected to the action of the flnids of the mouth, is anything but desirable as a material for filling teeth. Here we have two different kinds of action to contend with, to wit; *chemical* and *mechanical*; hence, one very important property to constitute a material for filling is that of *indistructibility*; a material, if possible, should have this power of resisting the action of surrounding influences.

Another essential property to constitute a material for filling is that *of adaptability*, by which is meant a capability of being wrought into suitable shape for purpose of protection; a facility of being applied and conformed to the parts against which it is placed. There are substances that would prove indestructible in the mouth, and that would be very desirable in many other respects, yet they are worthless for fillings, from the fact that they do not possess the property of being adapted to the parts. On the other hand, many substances possess adaptability, yet lack other very important qualities.

Another desirable property is *hardness*. A material may possess all other suitable qualities and lack this very *important* feature. Materials for fillings designed to occupy the grinding surface of the molars and bicuspids, should be hard enough not to be broken down or worn away during mastication. Again, a material should be as nearly as possible a nonconductor of heat, particularly for filling sensative teeth, or those liable to become so under the influence of slight causes. Great variations of temperatnre will in many instances aggravate sensitiveness, and in susceptible cases produce it, and if the irritation is continued *evil* results may attend. Gold, which possesses the largest number of desirable qualities as a material for filling teeth, is in this respect very defective; transmitting as it does every thermal change, immediately to the sensitive portion of the tooth. We may, however, remedy this defect by placing some non-conducting substance between the gold and the sensitive part. Among other properties mentioned, a material should be susceptible of being welded or united in a *solid* mass. The permanency of a filling depends largely upon this property. A filling having the various particles composing it perfectly *united* and *solid*, will prove *far* more durable than one in which the particles are not welded together, and are dependent one upon another for their retention. Mainly, because a *solid* filling is not so liable to become disintegrated, and broken down piece by piece. The operation of filling teeth is an interesting and important one, requiring great *care*, *skill*, and not a little experience.

Filling is the only means as yet ascertained of completely effecting the object for which it is employed, arresting decay and preserving the teeth. I do not deem it advisible to go into a discussion of the various metals that have been employed for filling teeth, as it is that which is the *best* that we are in search of. Besides it could not be anything more than a repetition of what has been said of them in the past. But of all the metals that have been used as materials for filling teeth gold stands preeminent, possessing as it does more than any other the requisite qualities for a good filling.

As to the form in which it should be used there is a diversity of opinion. Some operators claim advantages for one over another. You are all aware of the feeling that existed at one time in regard to the use of adhesive and non-adhesive gold ; and even at this time some are a *little* enthusiastic on the subject. But I think, (and no doubt you will bear me out in the assertion) that *better* results can be obtained by the use of that possessing the *adhesive* property.

In the use of soft or *non* adhesive gold, all depends upon the general form or shape of the cavity for its retention as a filling. On the other hand, in the use of the *adhesive* gold it is not so. It matters not what form or shape the cavity has, or whether you use the foil, the crystal, the block, the cylinder, or that in the form of pellets, the same results may be obtained. Not so much depends upon the form of the gold used as does upon the *manner* in which it is used. In filling teeth with gold the retention of the filling depends mainly upon secure anchorages, made by means of retaining pits found at the base of the cavity, and elsewhere where they can be made in firm and strong dentine. We may, however, in connection with the retaining pits, so form the cavity as to call into requisition the principle of wedging. But from this base or achorage the structure of the filling proceeds, and with almost the force necessary for the perfect condensation of the gold, directed in a line with the longitudinal axis of the tooth.

In making adhesive fillings the lateral force applied is light in comparison with the welding force required in making non-adhesive filling ; there is not that tendency to breaking the walls as where so much lateral pressure and wedging force is used ; while the filling can be made more *compact*, thereby giving greater durability. This method, I think, will clearly demonstrate to each one the advantages to be derived from its adoption.

There are cases in which the wedging method can not be employed ; as where it is impossible to so form a cavity so that it shall present two opposing walls paralell to each other ; or where the walls are so thin as to render sufficient wedging force impossible without liability of fracturing them, or placing them in danger of being easily broken down.

Now gentlemen, it would be simply absurd for us to advocate the filling every tooth with gold, or suggest any definite course. The surrounding circumstances must be taken into consideration.

The pathological conditions of the parts involved should be fully apprehended. Then use the means best adapted to the conditions. If we find that it would not be prudent to use the gold, we should use that which proximates closely.

There are several compounds that may serve a good purpose in the way of temporary fillings, among which are Guillois' cement, and Hill's stopping. But as you are well acquainted with them and their merits, I need not enumerate them.

As to the method of filling teeth with gold there are several. One favors the smooth points, another the serrated points. Many of our best operators use the serrated points exclusively. I think that the serrated point leaves a better receiving surface for the next piece of gold ; and if the serrations are very *fine* and *delicate* and great *care* is exercised in having each particle of gold perfectly condensed, the pittied surface so frequently found is not likely to exist when the filling is completed. The better plan, I think, is to have both the serrated and smooth points lying on the operating tray, then use such points as seem best in each case.

We may locate the retaining pits at the most favorable points. Care having been exercised in making them in firm and strong dentine, the margins of the cavity made smooth by the use of the smooth round file or the chisel, in order that we may be enabled to make as close a union as possible with the gold. If the cavity is in the proximal surface of the tooth, and there is not sufficient space between the teeth to admit of free and ready access, do not hesitate to employ the wedge. Above all things do not seek to gain admission by the use of the file. As this practice presents a very unsightly appearance, opens up a new avenue for the decay-producing agents, as well as destroying the *beauty* and *contour* of the organ thus operated upon.

Every thing now in readiness, the cavity *fully* prepared, steps are taken to keep the tooth perfectly dry. This very essential precaution is second to none other in the process of filling teeth. Without this we need not expect to accomplish the desired end ; and in order that we may do this effectually we must apply the *rubber dam.* True, a little time and labor may be expended in adjusting it, and the patient may suffer a little inconvience for a short time, but we will find ourselves amply rewarded when the work is completed.

Now wipe out the cavity, making it *perfectly dry* by means of a small piece of spunk ; or inject a current of *warm* air from the air syringe, made for the purpose. Commence in some remote corner or part of the cavity, filling one pit then another, building on, and locking each piece of gold firmly with another. Anneal each pellet of gold before introducing it into the cavity. Use the plugging pliers for the purpose of picking up the gold and passing it through the flame of the spirit lamp.

The practice with many of picking up the gold with the plugging point, and passing it through the flame also, is an objectionable one. In so doing the temper imparted to the instrument by the mannfacturer is drawn, the serrations become blunted, and many times destroyed. And not unfrequently the particles of gold are drawn from its place before it has been consolidated.

Place the particle of gold *just where you wish it to remain.* Consolidate each piece with the mallet in the hands of a good assistant. Continue in this manner until the cavity is full, adding surplus enough to enable you to make a good finish. I think it a good plan to use *heavy* gold at this point, say No. 30 or 60 cut in small pieces ; place the slip or piece where ever desired. Use the smooth "foot" instruments or the burnisher for thoroughly condensing the surface. I do not think that we can be too careful in packing the gold closely up to the walls of the cavity. Make it as it were a *part of the tooth.*

Care should be exercised in finishing not to allow the borders of the gold to project over the margins of the cavity, but simply to allow it to come up even with the surface of the enamel. When the gold is permitted to project over the margins of the cavity, fluids and foreign subtances are liable to be insinuated beneath, and the same difficulty may ensue that we have sought to overcome.

The general routine to be gone through with in finishing is familiar to all of you.

Gentlemen, these are *thoughts* suggested to me at time of writing. I am fully aware that they are not unknown to any of you, and yet I trust that they may meet with your hearty approval.

